# Ocular health practices by dental surgeons in Southern Nigeria

**DOI:** 10.1186/1472-6831-14-115

**Published:** 2014-09-11

**Authors:** Clement Chinedu Azodo, Ejike B Ezeja

**Affiliations:** Department of Periodontics, University of Benin, Benin City, Nigeria; Department of Preventive Dentistry, University of Benin Teaching Hospital, Benin City, Nigeria

**Keywords:** Professional eye examination, Safety eye goggle use, Self-rated ocular health, Dental surgeons, Dental procedures

## Abstract

**Background:**

Dental professionals are among the occupational groups that experience ocular injuries and problems as they perform their daily dental works. The purpose of the study was to determine the ocular health practices by dental surgeons in Southern Nigeria.

**Methods:**

This cross-sectional study was conducted on dental surgeons working in Southern Nigerian tertiary oral healthcare centers using self-developed validated questionnaire as the tool of data collection.

**Results:**

Of the 148 respondents, 27 (18.2%) rated their ocular health as poor/fair. More than half 82 (55.4%) of the respondents have undergone professional eye examination with a quarter 20 (24.3%) of them having received it, in the last 6 months. Symptomatic care was the major reason for the last visit. Medicated glasses use was found to be significantly associated with perception of ocular health and receipt of professional eye examination. A total of 32 (21.6%) and 2 (1.4%) of the respondents reported non-use of eye goggles and face mask respectively. Non-availability and associated visual clarity with goggle use were the main inhibitor to the regular safety eye goggles use among the respondents. The main suggested ways among the respondents of improving goggle use were training and provision of goggles free of charge for dental surgeons. Only 32 (21.6%) of the respondents would be uncomfortable reminding their colleagues on need to use safety eye goggle while attending to patients.

**Conclusion:**

Data from this study revealed that a significant proportion of the respondents rated the ocular health as excellent/good and do not regularly indulge in eye safety practices. Implementation of recommendation by the respondents may improve occupational eye safety among dental surgeons in Southern Nigeria.

## Background

In adaptation of definition of health by World Health Organization (WHO), ocular health is considered as a complete state of physical, social and mental well-being in relation to vision and not necessarily the absence of disease and infirmity [1]. Ocular health is becoming an increasingly important issue in both the healthcare sector and society as a whole because undetected and untreated ocular conditions can lead to vision loss and blindness. The role of the eyes in mobility, function, and enjoyment of life underscore the importance of maintaining good ocular health. Vision disorders has been cited as the fourth most prevalent class of disability in the United States [2] and the most prevalent handicapping conditions in childhood [3].

However, there are simple preventive and corrective measures to maintain good vision and consequent enjoyment of lifelong ocular health. These include wearing eye safety devices (safety glasses, protective goggles or face shields) while participating in sports or working with hazardous and airborne materials which lowers the risk of eye injury, damage to vision, and complete loss of sight. Visiting eye care professional for screening as early vision screening can lead to the detection, treatment, and prevention of many eye diseases. This is further illustrated by the fact that vision screening at well-child visits in medical homes significantly decreased the permanent visual loss due to amblyopia which is the medical term used when vision in one eye is reduced because the eye and the brain are not working together properly [4].

The prevalence of ocular problems among Nigerian of different ages arising from aging, traumatic, genetic, nutritional, environmental and occupational agents qualify it as a significant overlooked health area [5–7]. Dental professionals are among the occupational groups that experience ocular injuries and problems as they perform their daily dental works [8]. Studies in Nigeria [9] and Saudi Arabia [10] have consistently reported high prevalence of conjunctivitis, work-related ocular injuries and infrequent eye protective device use in dental practice. Forty-three (43.0) percent of orthodontists in United Kingdom reported instances of ocular injury in their practices with majority of them occurring during debonding or trimming acrylic [11]. Seventy three percent of Greek endodontists reported ocular accidents with amalgam and NaOCl being the foreign bodies most frequently associated with them [12]. Self-reported ocular deterioration and impairment have also been reported in Nigerian Dental Surgeons [13]. There is therefore a need to determine the ocular health practices by dental surgeons in Southern Nigeria. Stokes et al. [14] in New Zealand recommended proper eye protective procedures in dental practices more than two decades ago because of the random nature of many eye injuries. Dental surgeons should be concerned about their ocular health and safety and are expected to protect their eyes with safety glasses, face shields or goggles which are designed to protect against work-related occupational injuries from projectiles, chemicals, dust, heat and biohazards. Despite the values and importance of eye protection, there have been documented evidences of poor eye safety practices in Nigeria [15, 16]. The purpose of the study was to determine the ocular health practices by dental surgeons in Southern Nigeria.

## Methods

This was a cross-sectional survey of 185 selected dental surgeons working in Oral Healthcare Centres of University Teaching Hospitals in Southern Nigeria. The criterion for selection was absence of self-reported chronic medical conditions. These Teaching Hospitals are actively involved in undergraduate and postgraduate training of dental workforce and have all cadres of dental surgeons in their employment. The hospitals include University College Hospital Ibadan; University of Benin Teaching Hospital, Benin City; University of Nigeria Teaching Hospital, Enugu; University of Port Harcourt Teaching Hospital, Port Harcourt; Lagos University Teaching Hospital, Idi-Araba and Obafemi Awolowo University, Ile-Ife. The research protocol was reviewed and approved by the College of Medical Sciences, University of Benin, Benin-City, Nigeria Research and Ethics Committee. Informed consent was obtained from all the research participants. The survey was anonymous without identifiers. Participation was voluntary and no incentive was offered. A pretested self-administered questionnaire designed by the authors was used for data collection. The questionnaire was test and re-tested on ten dental surgeons working in private and secondary health facilities in Benin-City in a four week interval with reliability Cronbach's alpha of 0.85. The questionnaire elicited information on demography (age, gender, years of practice experience, medicated glasses use, and average number of patient treated daily), perception of ocular health, reasons and timing of eye care visit, safety eye goggle and face mask use, barriers to regular use and ways to improve regular goggle use. Data obtained were subjected to descriptive statistics in form of frequencies, percentages and cross tabulations using Statistical Package for Social Sciences (SPSS) version 17.0. Chi square test was employed to check statistical significance. P < 0.05 was considered significant at 95% confidence interval. For the purpose of analysis the age of respondents was categorized as ≤30 years and >30 years while and years of practice as ≤5 years and >5 years.

## Results

A total of 148 questionnaires were filled and returned, giving an overall response rate of 80.0% (148/185). Seventy nine respondents were less than 30 years old (53.4%), 87 (58.8%) were males, 110 had less or equal to 5 years practice experience (74.3%) and 85 treated an average of 1–3 patients per day (57.4%). About one-third 47 (31.8%) of the respondents use medicated glasses. The reason for medicated glasses use were myopia 30 (61.7%), hyperopia 5 (10.6%), astigmatism 12 (25.5%) and photophobia 1 (2.1%). A total of 27 (18.2%) rated their ocular health as poor/fair while 121 (81.8%) rated it as good/excellent. Perceived ocular health was not significantly associated with age, gender, years of practice and patient volume per day. However medication glasses use was significantly associated with perceived ocular health (Table 1). More than half 82 (55.4%) of the respondents had undergone professional eye examination. This was not significantly associated with age, gender, years of practice and patient volume per day. However medication glasses use was significantly associated with professional eye examination (Table 2). Of the 82 re-spondents that had professional eye examination, 20 (24.3%) had it in the last 6 months, 21 in the past 7–12 months (25.6%), 9 in the past 13–24 months (11%) and 32 more than 24 months ago (39%).

**Table 1:** **Association between demographic/professional data of the participants and perceived ocular health**

	Perception of ocular health			
	Poor/fair n (%)	Good/excellent n (%)	X ^2^	P-value
Characteristics				
Age (years)			1.22	0.270
≤30	17 (21.5)	62 (78.5)		
>30	10 (14.5)	59 (85.5)		
Gender			0.66	0.418
Male	14 (16.1)	73 (83.9)		
Female	13 (21.3)	48 (78.7)		
Year of practice			0.27	0.603
≤5	19 (17.3)	91 (82.7)		
>5	8 (21.1)	30 (78.9)		
Medicated glasses use			6.15	0.013*
Yes	14 (29.8)	33 (70.2)		
No	13 (12.9)	88 (87.1)		
Average number of patient treated per day			0.05	0.827
1-3	15 (17.6)	70 (82.4)		
>3	12 (19.0)	51 (81.0)		
Total	27 (18.2)	121 (81.8)		

(*) = statistically significant.

**Table 2:** **Associations between demographic/professional data of the participants and professional eye examination**

	Eye check			
	Yes n (%)	No n (%)	X ^2^	P-value
Characteristics				
Age (years)			0.84	0.358
<30	41 (51.9)	38 (48.1)		
>30	41 (59.4)	28 (40.6)		
Gender			0.16	0.686
Male	47 (54.0)	40 (46.0)		
Female	35 (57.4)	26 (42.6)		
Year of practice			2.23	0.135
≤5	57 (51.8)	53 (48.2)		
>5	25 (65.8)	13 (34.2)		
Medicated glasses use			55.43	0.001*
Yes	47 (100.0)	0 (0.0)		
No	35 (34.7)	66 (65.3)		
Average number of patient treated per day			0.49	0.484
1-3	45 (52.9)	40 (47.1)		
>3	37 (58.7)	26 (41.3)		
Total	82 (55.4)	66 (44.6)		

(*) = statistically significant.

Symptomatic care was the major reason for the last visit; routine check was the reason for ophthalmologic examination among 30 participants (36.6%) (Table 3). Care received by the respondents that were mainly eye glasses for non users 32 (39.0%), eye drops −16 (19.5%) and change of eye glasses for users 13 (15.9%) (Table 4). A total of 32 (21.6%) and 2 (1.4%) of the respondents reported non-use of eye goggles and face mask respectively (Table 5). Non availability [non provision of goggle by hospital authority 47 (31.8%) and not having personal goggle 30 (20.3%)], and disturbance of vision clarity 27 (18.2%) were the main inhibitors to the regular use of goggles. The main recommended ways by the respondents of improving goggle use were goggle provision at no cost 72 (48.6%), training dentists on the importance of goggles in ocular safety 37 (25.0%) and consideration of non-use as malpractice 20 (13.5%) (Table 6). Only 32 (21.6%) of the respondents would be uncomfortable reminding their colleagues on the need to use goggle while attending to patients. None and irregular eye protector wearers reported more poor/fair perceived ocular health than regular eye protector wearers and this was statistically significant. The reason for eye care visit was not statistically associated with perceived ocular health (Table 7).

**Table 3:** **Number (N) and percentages (%) of respondents in relation to the reasons for the last eye care visit**

Reason	Frequency (n)	Percent (%)
Routine check	30	36.6
Difficulty in reading	17	20.7
Redness of eye	10	12.2
Pain	9	11.0
Itching	5	6.1
Glass repair	2	2.4
Double vision	1	1.2
Others	8	9.8
Total	82	100.0

**Table 4:** **Eye care services received by the respondents**

Care	Frequency	Percent
	N	%
Eye glasses	32	39.0
Eye drops	16	19.5
Change of eye glasses	13	15.9
Tablets/or capsules	4	4.9
Eye surgery	2	2.4
Unspecified	15	18.3
Total	82	100.0

**Table 5:** **The frequency of utilization of eye protector and face mask among the respondents**

	Facial Barrier use	
	Eye protector n (%)	Face mask n (%)
Pattern of use		
Never	32 (21.6)	2 (1.4)
Irregular	66 (44.6)	8 (5.4)
Regular	50 (33.8)	138 (93.2)
Total	148 (100.0)	148 (100.0)

**Table 6:** **The recommended ways to improve goggle use among the respondents**

Ways	Frequency	Percent
Provision of goggle at no cost	72	48.7
Training dentists on the importance of goggle in ocular safety	37	25.0
Consideration of non-use as malpractice	20	13.5
Monitor of goggle use by hospital authority	16	10.8
Unspecified	3	2.0
Total	148	100.0

**Table 7:** **Association between perceived ocular health, eye protector wear and reason for eye care visit**

	Perception of ocular health				
Variable	Poor/fair n (%)	Excellent/good n (%)	Total n (%)	X ^2^	P-value
Eye protector wear					
Never	15 (46.9)	17 (53.1)	32 (100.0)	22.447	0.000*
Irregular	7 (10.6)	59 (89.4)	66 (100.0)		
Regular	5 (10.0)	45 (90.0)	50 (100.0)		
Reason for eye care visit					
Symptomatic	10 (19.2)	42 (80.8)	52 (100.0)	0.084	0.772
Preventive	5 (20.0)	25 (20.0)	30 (100.0)		

(*) = statistically significant.

## Discussion

The perception of ocular health which was modeled from the global oral and general health rating is expected to permit individuals to subjectively describe their health in epidemiological study. In this study, about one-fifth (19.3%) of the respondents rated their ocular health as fair/poor. The reasonable proportion of respondents with this rating implies this, as a health issue of concern. This indicates that assessment of clinical indicators of the poor ocular perception through ophthalmological examination may be necessary.

Vision problems constitute a substantial burden on the affected individuals, their caregivers, healthcare payers, and the national economy. Although regular comprehensive eye examinations are essential for prevention and timely treatment of eye disease to maintain ocular health, a previous study has shown that substantial percentages of persons do not seek eye care services, despite having visual impairment [2]. The perfidious onset of vision changes has led to the recommendation that all dentists should undergo eyesight testing at intervals of 2 years until the age of 50, and more frequently thereafter [17]. This is in accordance with that of the sight test intervals recommended by the Association of Optometrists (2006) [18] for the general population. It is well recognized that good eyesight is important for the practice of dentistry but it is apparent that regular eye testing are not undertaking by dentists [19]. In this study, more than half (55.7%) of the respondents had professional eye examination and 50% of them had it, in the last 12 months. This signifies that professional ocular examination was not a major health seeking behaviour by the respondents and portends that the recommended annual eye check-up was not well imbibed. This non-adherence to the recommended annual eye check-up has also been reported among doctors in Lagos, Nigeria but the prevalence of professional eye examination (86.0%) [20] was higher than the reported value in this study. Chadwick et al. [19] reported that only 16.0% of dentists in United Kingdom failed to seek routine eye examination at least every two years. A total of 36.6% of the respondents attended for the eye professional check in this study which is lower than the 57% reported in Scotland [21] and the 54% reported amongst Greek endodontists [12]. This may imply that dentists in developing countries are less concerned about their ocular health than dentists in developed countries. The symptomatic care was the major reason for the last visit in this study signifying that the regular eye examinations should be encouraged. This form of eye care visit explained the pattern of care received by the respondents which were eye glasses prescription, eye surgeries and medications in form of tablets, capsules and eye drops.

In this study, about one-third (31.8%) of the respondents use medicated glasses which is comparable to 36.1% reported among dental surgeons attending continuing education course in Nigeria [13]. The medicated glasses use is significantly associated with the prevalence of eye care visit and perception of ocular health. This tallied with the findings among dentists in the United Kingdom with known eyesight deficiencies attending for more routine eye examination than their counterparts [19]. Also dental students who had their eyesight corrected were significantly more likely to attend for examination biennially than those without correction [22]. The higher poor/fair ocular health rating among medicated glass users may not be unconnected with the psychology of prosthesis to augment the physiological function of organs or tissues of the body. This is confirmed by the report of a previous study which stated that wearing eyeglasses can negatively affect physical self-esteem [23]. Similarly wearing oral prosthesis in form of removable denture resulted in poor self-rated oral health [24].

Eye safety device use in form of safety glasses and protective goggles while working protects against foreign bodies, splashes, aerosols, curing light, projectiles, chemicals, dust, heat and biohazards thereby lowering the risk for eye injury and damage to vision. Eye safety practices are included in the Guidelines for Infection Control in Dental Health–Care Setting 2003. The adoption of protective eyewear has been found to patchy thereby exposing dentists to unnecessary risk [19]. Although eye protection are categorized into adequate and inadequate with adequate eye protection including prescription glasses with side shields, face shields, safety glasses and magnifying loupes and inadequate eye protection including prescription glasses without side shields and without adequate frame diameter [25]. However protective eye wear in this study was categorized into regular, irregular and none use as previously used eye safety practices studies among dental healthcare providers in Nigeria. In this study, protective eye wear was regular (33.8%) and irregular (44.6%) among the respondents. It was also found that 21.6% of the respondents never used safety eye goggle. The relative higher regular use of face mask than eye goggle [93.2% versus 33.8%] reflects the non-availability and associated discomfort with eye goggle use. The highly visual demanding work performed by dentists requires safety glasses that are not yet available on the market, which might also explain the none and irregular use [26]. Irregular eye protector wearers reported statistically more poor/fair perceived ocular health than regular eye protector wearers explaining that preventive health behaviour results in better health. The predominantly cited barrier to safety eye goggle use were non-availability and disturbance of vision clarity. The fact that non-availability and disturbance of vision were the predominant reasons for non goggle use explained why respondents recommended training and provision of goggles free of charge as the main ways of improving safety eye goggle use. It was also revealed from this study that peer model may facilitate improvement in the use of goggle as 78.4% of the respondents would be comfortable reminding their colleagues on the need to use goggle while attending to patients. It has been shown that improvement in the utilization of eye care services occurred among Australians who were not aware of eye health and needed routine eye examinations after health promotion by primary care providers [27]. Dentists therefore should be encouraged to be actively involved in their ocular health in collaboration with eye care professional inorder to increase their chances for achieving and maintaining good ocular health.

## Conclusion

Data from this study revealed that a significant proportion of the respondents rated the health of their eyes as excellent/good. The symptomatic care was the major reason for professional eyecare visit and appreciable proportion of the respondents does not regularly indulge in eye safety practices. Implementation of recommendation of the respondents would improve occupational eye safety practices among dental surgeons in southern Nigeria.

## Authors’ information

CCA holds Bachelor of Dental Surgery (BDS) Degree, Master of Physiology (MSc Physio.) degree, Master of Public Health (MPH) degree and Fellowship of National Postgraduate College of Nigeria (FMCDS) lectures Periodontology in University of Benin and practices as a Consultant Periodontology at the University of Benin Teaching Hospital Benin City, Nigeria.

EBE holds Bachelor of Dental Surgery (BDS) Degree and is currently a DDS students of Minnesota Dental School, United States of America.
